# Cardiopulmonary Complications of Klippel-Feil Syndrome: A Case Report

**DOI:** 10.7759/cureus.67303

**Published:** 2024-08-20

**Authors:** Ranjiv-a M Bridglal

**Affiliations:** 1 Faculty of Medical Sciences, The University of the West Indies, St. Augustine, TTO

**Keywords:** klippel-feil syndrome, congenital synostosis, pulmonary hypertension, cervical vertebrae fusion, ventricular trigeminy and bigeminy

## Abstract

Klippel-Feil syndrome is characterized by the congenital synostosis of multiple cervical vertebrae and commonly presents with a multitude of congenital abnormalities, mainly including cardiac and respiratory defects. We present the case of a 39-year-old male with a prolonged history of cardiopulmonary symptoms whose investigations revealed restrictive lung disease, ventricular trigeminy and bigeminy, cervical vertebrae fusion, thoracic lumbar scoliosis, and mild-to-moderate pulmonary hypertension, all consistent with a diagnosis of Klippel-Feil syndrome. His management focused on preventing the progression of these findings while minimizing the effects of his extrinsic pulmonary restriction. Given the lack of guidelines in the management of such patients, this report highlights the role of early diagnosis and adequate management of such patients to reduce its progression and prevent the development of complications.

## Introduction

Klippel-Feil syndrome, also known as cervical vertebra fusion syndrome, was first described by Maurice Klippel and Andre Feil in 1912 and includes at least one of the following major features: a short neck, a low hairline at the back of the neck, and a limited range of motion of the neck. It is characterized by the synostosis of two or more cervical vertebrae which is present from birth and is associated with genetic mutations that are responsible for proper bone development [1]. Such mutations include the *GDF6*, *GDF3*, or *MEOX1* genes [1]. MOX-1 is a protein encoded by the *MEOX1* gene and controls the sequence of events involved in the parting of vertebrae from one another [1]. The deficiency of this protein can result in the failure of separation of any vertebrae in the spine [1]. Hence, this syndrome is clinically related to congenital scoliosis and Sprengel’s deformity, i.e., elevated scapula [1]. Given the limited cervical range of motion due to the associated vertebral fusions seen in Klippel-Feil syndrome, coupled with resultant scoliosis, securing airway access in emergency and surgical, i.e., anesthetic, settings may prove to be challenging [2]. This is an important consideration when expectant management fails to control the patient’s symptoms and when the patient deteriorates acutely and requires respiratory support [2].

Patients born with this syndrome may have resultant congenital heart defects [2]. These include ventricular septal defects, coarctation of the aorta, complete heart block, atrial septal defects, total anomalous venous connection, and aortic regurgitation [2]. Perfusion-ventilation mismatches, reduced ventilation turnover, and decreased expiratory flow all result from diminished respiratory mechanisms [3]. This results in pulmonary hypertension and type 2 respiratory failure, which has been linked to the restrictive effects induced by scoliosis [3]. Patients with this syndrome may also have congenital mandibular hypoplasia and cleft palate, which has been linked to upper airway obstruction and, consequently, obstructive sleep apnea [2].

Patients with Klippel-Feil syndrome require lifelong care, especially to monitor for life-threatening complications. Upon a literature review, there are no published reports on Klippel-Feil syndrome, nor its associated cardiac and respiratory complications in Trinidad and Tobago. We describe a case of Klippel-Feil syndrome which presented at a private institution in Trinidad and Tobago and its associated cardiac and respiratory complications.

## Case presentation

A 39-year-old male, a welder, presented with a three-year history of non-radiating chest pains, palpitations, and orthopnea despite the use of budesonide/formoterol. He had a Medical Research Council dyspnea score of 2, i.e., walking at a slower pace and stopping for breath when walking at ground level. It was accompanied by an occasional dry cough which had no diurnal variation. He also reported daytime drowsiness and snoring. His medical history was significant for asthma, penicillin allergies, and a left-sided inguinal hernia. His mother was prescribed and compliant with an antiemetic, containing doxylamine, dicyclomine, and pyridoxine, while pregnant with him for an unknown duration, i.e., from 1982 to 1983, early in the pregnancy. His family history was significant for prostate cancer and hypertension, but, of note, no diagnosed genetic disorders.

On initial evaluation, his vital signs were the following: heart rate of 64 beats/minute, blood pressure of 120/80 mmHg, and oxygen saturation of 98% on room air. His body mass index was 31.9 kg/m^2^ (weight was 81.6 kg and height was 160 cm), and his neck size was 43.18 cm. Physical examination revealed chest wall abnormalities consistent with pectus excavatum, i.e., sunken sternum and asymmetrical rib distribution, and, bilaterally, clear lungs on auscultation with good air entry. Other systemic examinations, including cardiovascular examination, were consistent with normal findings.

Blood investigations including complete blood count, metabolic profiles (renal, liver, and thyroid function tests), and immunologic testing were within normal ranges (Table 1).

**Table 1:** Initial laboratory investigations. WBC = white blood cell count; HBG = hemoglobin; PLT = platelets; ESR = erythrocyte sedimentation rate; BUN = blood urea nitrogen; Cr = creatinine; eGFR = estimated glomerular filtration rate; AST = aspartate aminotransferase; ALT = alanine transaminase; ALP = alkaline phosphatase; HbA1C = glycated hemoglobin; Ca = calcium; Mg = magnesium; P = phosphorus; Alb = albumin; TSH = thyroid-stimulating hormone; FT4 = free T4; CRP = C-reactive protein

Parameter	Units	Result(s)	Reference range
Hematology			
WBC	10^3^/μL	5.8	4.0–10.5
HBG	g/dL	14.5	11.5–16.5
PLT	10^3^/μL	189.0	150–400
ESR	mm/hour	5.0	0.0–15.0
Biochemistry			
BUN	mg/dL	10.6	6–20
Cr	mg/dL	0.86	0.5–1.2
eGFR	mL/minute/1.73m^2^	127.49	>90.0
AST	U/L	21.6	5.0–34.0
ALT	U/L	29.4	0.0–55.0
ALP	U/L	48.4	40–129
HbA1C	%	6.1	4.0–6.4
Ca	mg/dL	9.2	8.4–10.2
Mg	mg/dL	1.98	1.6–2.6
P	mg/dL	2.30	2.3–4.7
Alb	g/dL	44.0	3.5–5.0
Uric acid	mg/dL	6.1	3.5–7.2
TSH	μIU/mL	0.854	0.4–4.0
FT4	ng/dL	1.32	0.89–1.76
CRP	mg/L	1.60	0.0–5.0
Immunology			
Total IgE	IU/mL	31.80	<100

Resting ECG showed ventricular bigeminy, and a 24-hour Holter monitor revealed a ventricular ectopic burden of 28% with monomorphic ventricular trigeminy and ventricular bigeminy throughout most of the study. Echocardiography illustrated an ejection fraction of 68%, and mild-to-moderate pulmonary hypertension (right ventricular systolic pressure of 48 mmHg). The coronary angiogram was consistent with normal findings and computed tomography of the chest, abdomen, and pelvis showed curvature to the left of thoracic lumbar scoliosis and fusion of C4/C5/C7/T1/T2/T3/T4.

Fractional exhaled nitric oxide testing was within normal limits. Pulmonary function testing (PFT) showed the following findings: mild restrictive ventilatory defect, moderate gas transfer defect, and normal diffusion capacity of the lung for carbon monoxide/alveolar ventilation (transfer coefficient for diffusion of CO into the blood) (Table 2).

**Table 2:** Initial baseline pulmonary function test results comparing response to bronchodilator (Salbutamol). FVC = forced vital capacity; FEV = forced expiratory volume; TLC = total lung capacity; RV = residual volume; DLCO = diffusion capacity of the lung for carbon monoxide; VA = alveolar ventilation

Parameter	Units	Result(s)		Change (%)
		Without bronchodilator	With Bronchodilator	
Spirometry				
FVC	L	2.52	2.39	-5
FEV_1_	L	2.09	2.07	-1
FEV_1_/FVC	%	83	87	5
Lung volumes (He)				
TLC	L	4.14	-	-
RV	L	1.57	-	-
RV/TLC	%	38	-	-
Diffusing capacity				
DLCO (Hb)	mL/minute/mmHg	17.51	-	-
DLCO/VA	mL/minute/mmHg/L	4.89	-	-

Following these investigations, his diagnoses included Klippel-Feil syndrome with ventricular bigeminy, thoracic lumbar scoliosis, and mild-to-moderate pulmonary hypertension. He was continued on budesonide/formoterol and consideration will be given to starting a trial of tiotropium bromide.

Following three weeks of steroid and muscle relaxant therapy, the patient reported that he was still experiencing exertional dyspnea and orthopnea and had not improved or worsened. He was considered clinically stable and scheduled to have a follow-up PFT and echocardiogram to monitor his ventilatory defect and pulmonary hypertension, respectively. The patient was counseled on the need for and importance of flu and pneumonia vaccinations for the prevention of pneumonia to which he complied, as well as the role of pulmonary rehabilitation exercises. The patient was also advised on the role of arterial blood gas evaluation in diagnosing respiratory failure.

## Discussion

The clinical and radiological diagnosis of Klippel-Feil syndrome in this patient was consistent with previously diagnosed cases, i.e., he had fusion of the two cervical vertebrae together, C4 and C5, as well as the fusion of C7 to T4. A single-level congenitally fused cervical vertebrae constitutes type I, while type II constitutes several fused vertebrae that are non-contiguous [1]. Type III entails multiple continuous fused vertebrae. This patient presented with type III, i.e., the fusion of C4/C5 and C7/T1/T2/T3/T4 [1]. Another characteristic congenital malformation of Klippel-Feil syndrome is Sprengel deformity, the unilateral elevation of the scapula, which was not evident in this patient, but is considered the second most common association with this syndrome [1].

Structural cardiac defects are the more frequent cardiac abnormalities associated with this syndrome [4]. However, our patient presented with ventricular irritability (ventricular bigeminy and trigeminy), which may be due to infiltration or fibrosis of the myocardium.

Given that this is a congenital malformation, untreated early-onset scoliosis may lead to exacerbated spinal deformities, and, consequently, restrictive lung disease may manifest, which was present in this patient (decreased total lung capacity with preserved forced expiratory volume in one second/forced vital capacity) [2]. The patient’s diagnostic workup also revealed pulmonary hypertension, which is a documented complication of this condition and can be the etiology of the patient’s exertional dyspnea and orthopnea [5]. Therefore, the aforementioned factors categorized this patient as having group 3 pulmonary hypertension, chronic lung diseases [5]. Management of the underlying causes of pulmonary hypertension is key in preventing progression. The patient’s extrinsic pulmonary restriction, i.e., spinal deformity cannot be structurally changed, and, hence, medical management was used as corticosteroids to reduce inflammation and increase airflow [6]. Pulmonary rehabilitation may play a crucial role in preventing the progression of the severity of pulmonary hypertension, allowing improvements in oxygen intake, reduction of exertional dyspnea, and increased inspiratory muscle strength, all of which may prove beneficial to the patient [7]. Given that no significant structural abnormalities were revealed by the echocardiogram, the patient’s alternating normal sinus and premature ventricular complexes, i.e., ventricular bigeminy may be explained by potential hypoxemia created by the hypoventilation caused by the restrictive lung disease pattern; however, there was no evidence of this in the patient [8].

Doxylamine, dicyclomine, and pyridoxine, an antiemetic, have been anecdotally linked to cases of congenital malformations among pregnant mothers who used them, but no teratogenic outcomes have been proven [9]. While this report was unable to pharmacologically link this drug to such malformations, it highlights its consequential use during the pregnancy of a patient born with Klippel-Feil syndrome. Its use during early pregnancy in this case is highly suspicious given the outcome of this syndrome and the diagnosis of Klippel-Feil syndrome was extrapolated based on the suspicion of being an etiological cause [9].

Limitations

This patient would have benefitted from a cardiac MRI to detect myocardial edema, infiltration, or fibrosis as the causative agent of his ventricular irritability. Further, due to the high ventricular ectopic burden, he should have also undergone electrophysiology studies and ablation [10]; however, he was unable to afford these procedures and was managed on guideline-directed medical therapy. Right heart catheterization with reversibility testing is the gold standard for the diagnosis of pulmonary hypertension but was not done in this case due to the patient’s financial constraints [11]. The same reason holds for the lack of polysomnography analysis, i.e., to determine the extent, if any, of obstructive sleep apnea, given its prevalence among such patients. Obstructive sleep apnea is an established cause of pulmonary hypertension with intermittent hypoxia leading to increased left atrial pressures and pulmonary venous hypertension, which further precipitates vascular remodeling and pulmonary arterial hypertension [11].

Given that arterial blood gas measurement is the gold standard for the diagnosis of respiratory failure, which is prevalent among patients with pulmonary hypertension, the patient was counseled on the importance of monitoring for such failure but he could not afford it [11]. Given that this patient presented with thoracic and lumbar scoliosis, it is possible that these skeletal abnormalities resulted in inefficient chest wall muscle use and, consequently, a diminished expiratory drive and hyperinflation. This inadequate ventilatory drive would result in a ventilation-perfusion mismatch, which invariably leads to hypercapnic respiratory failure [12]. Consequently, this patient’s diagnosis was made via clinical and limited radiological diagnoses, coupled with the antenatal use of doxylamine, dicyclomine, and pyridoxine.

## Conclusions

We present the first documented case of ventricular irritability, restrictive lung disease, and subsequent pulmonary hypertension in a patient with Klippel-Feil syndrome. This report presented rare and previously poorly documented cardiopulmonary complications of Klippel-Feil syndrome, its clinical manifestations, as well as the diagnostic workup and subsequent management of these complications while acknowledging the lack of advanced, invasive testing for this patient and its clinical value. Our patient, whose diagnoses were made using basic, minimally invasive testing modalities, demonstrated no clinical improvements following steroid and muscle relaxant therapy. Hence, pulmonary rehabilitation was discussed with the patient.

Cardiopulmonary complications in such patients can be consequential; hence, early detection and management using a multidisciplinary approach to these complications is essential to prevent their development and progression. We hope this stimulates further research into the complications of Klippel-Feil syndrome with emphasis on the diagnosis and management, as well as potential screening options.
